# Functionalized Polypropylene Copolymers as Multisubstrate Hot-Melt Adhesives

**DOI:** 10.1021/acsami.5c07594

**Published:** 2025-06-03

**Authors:** Alexander Evans, Clement G. Collins Rice, Zoë R. Turner, Dermot O’Hare

**Affiliations:** Chemistry Research Laboratory, Department of Chemistry, 6396 University of Oxford, 12 Mansfield Road, Oxford OX1 3TA, U.K.

**Keywords:** multisubstrate, hot-melt adhesive, polypropylene, rheology, lap shear strength

## Abstract

Amine-modified polypropylenes (**PP**_**R**_, R = NH­(Et)_2_ (DEA); NH­(Et)­(CH_2_CH_2_OH) (EAE); NH­(CH_2_CH_2_OH)_2_ (DEOA)) have been prepared via a two-step synthesis and display enhanced adhesive performance with respect to both steel and polypropylene (PP) substrates. PP typically displays poor adhesion to polar substrates, which consequently restricts its utility as a hot-melt adhesive (HMA). Solvent-free, quantitative postmodification of poly­(propylene)-*co*-(11-bromo-1-undecene) (range of comonomer incorporations (3–9 mol %)) with secondary amines yielded amine-modified PPs: **PP**_**DEA**_, **PP**_**EAE**_, and **PP**_**DEOA**_. Rheological and FT-IR characterization identified the presence of a PP-based supramolecular hydrogen bonding network. (Co)­polymers were evaluated as HMAs by lap shear strength between steel–steel and steel–plastic substrates. **PP**_**EAE**_ and **PP**_**DEOA**_ both excelled as HMAs between steel, recording the largest mean adhesive forces of 16.8 and 17.4 MPa, respectively; **PP**_**DEOA**_ displayed a 252-fold increase vs **PP** and comparable adhesive strengths to conventional structural adhesives. The adhesive failure mode in multisubstrate adhesion was found to be a function of interfacial effects, depending on the relative ability of the HMAs to bind to the polar steel surface and diffuse into the plastic substrate. **PP**_**DEA**_ and **PP**_**EAE**_ were found to be optimal in this case with failure by stock break indicative of adhesion greater than the tensile strength of the substrate and consequently appropriate for the application. The unique properties of these bifunctional materials highlight the versatility of the relatively limited application of functionalized PPs to date. This study now allows further sets of functionalized PPs to be readily prepared to meet a diverse array of multisubstrate adhesive requirements.

## Graphical abstract

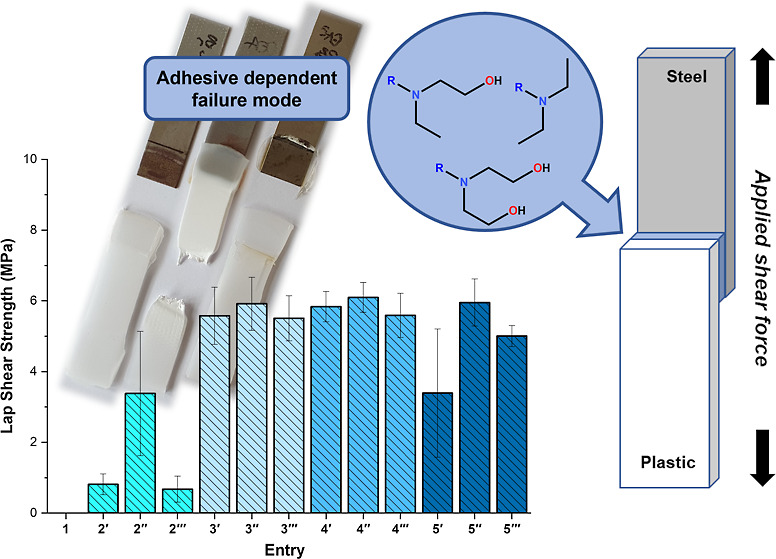

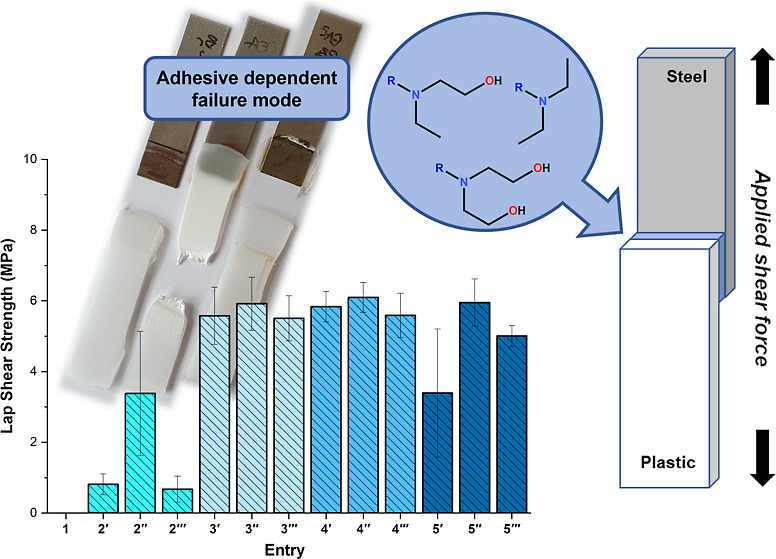

## Introduction

Adhesives are vital in everyday life, with widespread applications in construction, automotive, and consumer packaging, using over 6 billion lbs annually yet evidencing volume growth for high-performance adhesives.^1–5^ Consequential to an industry shift toward environmentally friendly, solvent- and volatile organic compound (VOC)-free adhesives,^6–10^ hot-melt adhesives (HMAs) are gaining prominence as they satisfy market needs for both ecological responsibility and strong product performance.^11–15^ Current adhesive strengths of commercially available ethylene vinyl acetate or polyurethane-based HMAs are limited to 6–7 MPa.^16^ Meeting demands for lightweight, high-performance products necessitates superior adhesion to diverse materials: plastics, metals, and wood.

Numerous adhesive solutions utilize polar polymers; however, bonding polyolefins (POs) to polar substrates is challenging due to their low surface free energy, chemical inertness, and absence of functional groups.^17–19^ Enhancing substrate adhesion often involves chemically or physically treating PO surfaces for increased polarity and improved wettability, accompanied by associated limitations such as limited control and functionality.^20–23^ Bonding to both high (metals) and low (plastic) surface free energy substrates, termed multisubstrate adhesion (MSA), presents a unique challenge. MSA through the use of HMAs has gained traction as an alternative to conventional mechanical and adhesive bonding methods. Unlike mechanical fastening, which can add weight, damage, and weaken the material, or standard adhesive joints requiring surface pretreatment and curing time,^24–26^ HMAs provide a fast, effective, and damage-free alternative. As such, there are numerous potential commercial applications for functional PPs as hot-melt adhesives, particularly as lightweight alternatives to traditional fastening methods in the automotive industry. Ultimately, such effective lightweight hot-melt adhesive joints can further contribute to the amelioration of carbon emissions.

POs have the potential to become attractive materials for adhesive applications because of their low density, low production costs, and tunable material properties. In particular, the nonpolarity and highly crystalline nature of isotactic PP are generally considered hallmarks of poor adhesives that have the potential to hinder its application in multimaterial assemblies. Surface modifications and functionalized adhesives have been developed to overcome these limitations by enhancing polymer interfacial compatibility with other materials, particularly metals.^16,27–29^ Ongoing research focuses on advanced functional polymers to improve adhesion to both polar and nonpolar substrates.^11,14,28^ Recently reported work by Duchateau and co-workers highlighted the application of propylene-based hydroxyl-functionalized terpolymers for use as HMAs with improved adhesive performance (12.6 MPa) at low application temperatures (130 °C) despite minimal incorporation levels (≤0.5 mol %);^16^ however, direct copolymerization as a method to access these materials is likely to have a limited range of functionality that can be incorporated. As an alternative to improving the polymer wetting, focus has likewise been directed at enhancing the wettability and bonding strength of the metal substrate, with recent work evidencing superior joint strengths up to 25 MPa by creating a surface texture that complements the adhesive properties of polymers.^25,26^

An additional complexity in polymer–metal bonding lies in the interphase, the three-dimensional boundary region where two materials meet.^30–34^ Understanding the interphase is critical for predicting and enhancing adhesive performance from an industrial perspective.^30,31,35,36^ Advanced techniques like sum frequency generation (SFG) vibrational spectroscopy have facilitated nondestructive analysis of these buried interfacial regions, revealing details about hydrogen bonding, acid–base interactions, and other molecular behaviors that contribute to interfacial adhesion strength.^37,38^ Nishino et al. recently described improved isotactic PP-rubber adhesion stemming from crystalline growth and the formation of lamellae interlock in the interphase region.^27^ Functional PP materials therefore offer a unique opportunity, able to provide binding sites to the metallic substrate while adhering to plastic substrates through the diffusion of molecular chains at the interface. The use of HMAs for multisubstrate applications provides an innovative approach to lightweight construction. By addressing interfacial challenges between metals and polymers, HMAs can enable the production of reliable, high-strength hybrid components for structural and functional parts in various sectors, such as construction and transport.

We now build upon our recent report of the synthesis of a set of low molecular weight amine- and amino alcohol-functionalized polypropylenes, prepared using a two-step copolymerization and postfunctionalization methodology, which were utilized as HMAs for steel–steel adhesion in preliminary studies.^29^ Herein, we synthesize and characterize a library of amine-modified polypropylene-based copolymers, spanning different incorporation levels and molecular weights, allowing for effective assessment of their adhesion as HMAs to steel and commercial PP. In particular, their utility as multisubstrate adhesives is highlighted as a unique example of interfacial adhesion, offering a solution to widespread industrial limitations with conventional multisubstrate adhesive systems.

## Materials and Methods

### Commercially Supplied Materials

11-Bromo-1-undecene (**11-Br**, Sigma-Aldrich) was dried over preactivated 3 Å molecular sieves, filtered, and freeze–pump–thaw degassed before use. Propylene (N2.5) was supplied by BOC Ltd. and was used as received. MAO was supplied by Chemtura Corporation as a slurry in toluene which was dried under vacuum before use. Dimethylsilylene bis­(2-methyl-4-phenyl-1-indenyl) zirconium­(IV) dichloride was used as supplied by SCG Chemicals plc. Diethylamine, ethyl­(ethanol)­amine, diethanol amine, 1,1,2,2-tetrachloroethane-*d*_2_, and triisobutylaluminum were used as received from Sigma-Aldrich. Plastic sheets were supplied from SCG Chemicals plc (P722JO) and are a polypropylene impact copolymer resin. Steel sheets (QD23.5, smooth mill finish, Q-Panel) were employed as surfaces for adhesive experiments from Q-LAB.

### Preparation of Functionalized Polypropylenes

The solution-phase copolymerization of propylene and **11-Br**, and all subsequent postpolymerization modifications, were carried out as previously reported.^29^

### Polymer Characterization

Rheology was performed on a TA Instruments Discovery HR-2 hybrid rheometer by using a temperature-controlled stainless steel Peltier plate and a flat parallel plate geometry (20 mm diameter) with a working gap of 1000 μm. Approximately 180 mg sample polymer was pressed using a pellet press under 10 tons of pressure which was then placed on the rheometer plate at 25 °C. Small amplitude oscillatory shear (SAOS) measurements were performed at 180 °C, soaked for 300 s prior to start of the test, under a flow of dry nitrogen in continuous oscillation (direct strain) mode at a strain of 1% and a logarithmic frequency sweep was performed from 0.01 to 100 rad s^–1^.

Fourier-Transform Infrared Spectroscopy spectra were collected on a Bruker VERTEX 80 FT-IR spectrometer fitted with a DuraSamplIR Diamond ATR. Before the sample scans, 128 scans were taken as a background over the range 4000–400 cm^–1^. Transmittance was recorded at a resolution of 4 cm^–1^ over the range 4000–400 cm^–1^ for 128 scans.

Lap shear strength samples were prepared via compression molding of the polymer. Propylene-based copolymers were loaded between the substrates: Steel QD35 and plastic, with an overlap of 10 mm (127 mm^2^ bonding area). Then, the compression–heating cycle was applied: (i) heating to 180 °C, (ii) stabilizing for 5 min with no force applied, (iii) applying for 5 min with 25 kN normal force and cooling down to 40 °C under 25 kN normal force. Before measurements, samples were conditioned for 7 days at room temperature. The measurements were performed using an Instron 5582 tensile tester equipped with a 5 kN load cell. The tests were performed on specimens (51 cm × 12.7 cm) with surface overlapping 10 mm at room temperature. A grip-to-grip separation of 140 mm was used. The samples were prestressed to 3 N and then loaded with a constant cross-head speed of 100 mm/min. To calculate the lap shear strength, the reported force value was divided by the bonding surface (127 mm^2^) of the specimens. The reported values are an average of at least 4 measurements of each composition.

## Results and Discussion

### Synthesis and Characterization of Functionalized Polypropylenes

The synthesis of brominated polypropylene copolymer, poly­(propylene)-*co*-(11-bromo-1-undecene) (**PP**_**Br**_) by solution-phase copolymerization of propylene and 11-bromo-1-undecene (**11-Br**), and subsequent transformations by diethylamine (DEA), 2-(ethylamino)­ethanol (EAE), and 2,2′-iminodiethanol (DEOA) resulted in the formation of **PP**_**DEA**_, **PP**_**EAE**_, and **PP**_**DEOA**_, respectively, as previously reported (Scheme 1 and Table 1).^29,39^

**Scheme 1:**
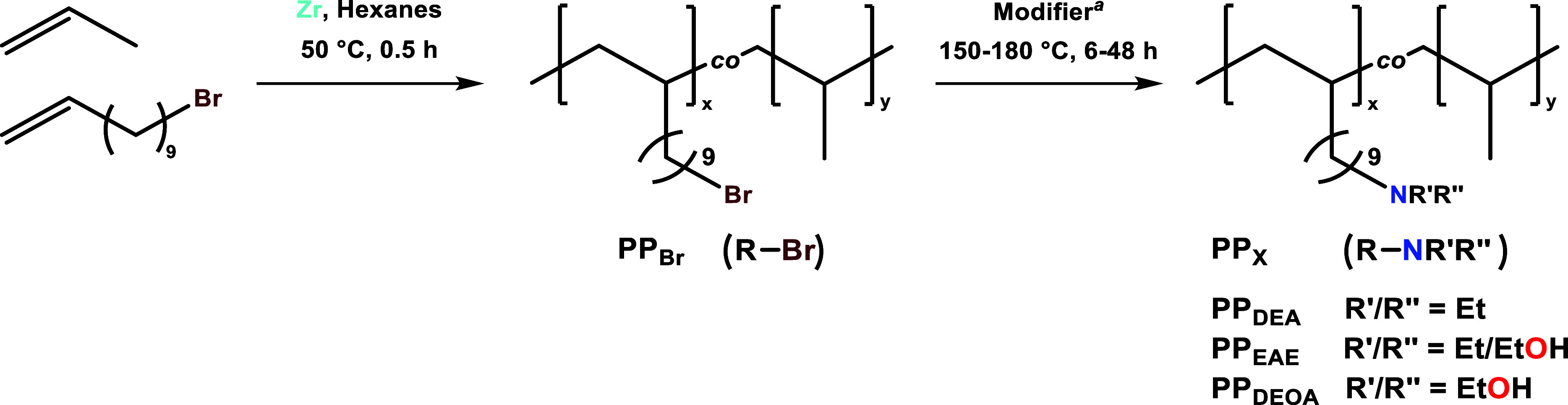
Synthesis of **PP**_**Br**_ by Solution-Phase Copolymerization^a^

a Zr = dimethylsilylene bis­(2-methyl-4-phenyl-1-indenyl) zirconium­(IV) dichloride ([Zr] = 0.024 mM), [**11-Br**] = 91, 137, or 182 mM yielding comonomer incorporation of 2.96, 4.78, and 9.10 mol %, respectively, as determined by ^1^H NMR spectroscopy. ^a^Modifier = DEA, EAE, or DEOA.

**Table 1:** (Co)­polymerization of Propylene (and 11-Bromo-1-undecene)^a^

entry	[**11-Br**] (mM)	activity^b^	productivity^c^	yield (g)	incorporation (mol %)^d^	*M*_w_ (kg mol^–1^)^e^	*D̵*_M_ ^e^
1‴	0	10,100	32.1	8.03	0.00	98	4.7
2‴	91	3580	11.4	4.27	2.96	226	2.8
2‴	137	2090	6.65	2.49	4.78	172	2.7
2‴	182	1230	3.92	1.47	9.10	163	2.5

a Conditions: dimethylsilylene bis­(2-methyl-4-phenyl-1-indenyl) zirconium dichloride (Zr), MAO used as a cocatalyst and scavenger with [Al]_0_:[Zr]_0_ = 1000:1. Molar ratio of [TIBA]_0_:[**11-Br**]_0_ = 1:10, 0.5 mL toluene, 49.5 mL hexanes, propylene 2 bar, polymerization temperature = 50 °C, 0.5 h. Polymerization was performed at least in duplicate and mean values reported.

b kg_PP_ mol_Zr_^–1^ h^–1^ bar^–1^.

c kgPP g_Zr_^–1^ h^–1^.

d Determined by ^1^H NMR spectroscopy.

e Determined by SEC using the Mark–Houwink correction for PP.

Our previously reported inchoate studies utilized low molar mass (*M*_n_) functionalized PPs (ca. 4000 g mol^–1^) produced by *rac*-ethylenebisindenyl zirconium­(IV) dichloride as hot-melt adhesives.^29^ Unfortunately, the predominant failure mode during lap shear strength analysis was cohesive failure as opposed to interfacial failure. This was attributed to the low *M*_n_ of the PPs resulting in a low ultimate tensile strength, less than that of the adhesive interfacial strength. Consequently, it was hypothesized that synthesizing high *M*_n_ analogues, with increased tensile strength, would enable a thorough assessment of the materials as hot-melt adhesives. The synthesis of **PP**_**Br**_ copolymers with increased *M*_n_ was achieved using dimethylsilylene bis­(2-methyl-4-phenyl-1-indenyl) zirconium­(IV) dichloride (Zr); molar mass was determined by size exclusion chromatography (*M*_n, SEC_ = 81,400–63,500 g mol^–1^; Table 1 and Figure S1). The range of **PP**_**Br**_ copolymers was achieved by varying [**11-Br**] (91 mM to 182 mM), eliciting differences in incorporation to evaluate the degree to which polar functionality contributes to adhesion. **PP**_**Br**_ copolymers containing 2.96, 4.78, and 9.10 mol % incorporation of **11-Br**, as determined by ^1^H NMR spectroscopy, were isolated and all subsequently functionalized with one of DEA, EAE, or DEOA and characterized (Table 2 and Figures S1–S6). The presence of the comonomer branches, in addition to the modifier R groups, generally suppresses crystallinity, leading to reduced melt temperatures (*T*_m_) and crystallinity with respect to **PP**. Since the Mark–Houwink correlation is not known for functionalized PP, and the presence of a polar and hydrogen-bondable group is likely to affect the radius of gyration in solution, SEC was not obtained for the amine-modified polymers, but it is assumed that the backbone chain length is unchanged during postpolymerization modification.^29,40–44^

**Table 2:** Characterization and Adhesive Data for Propylene (Co)­polymers

entry	modifier	incorporation^a^ (mol %)	*T*_m_^b^ (°C)	crystallinity^b^ (%)	*G*′^c^ (kPa)	*G*″^c^ (kPa)	|η*|^c^ (kPa·s)	steel plastic^d^ (MPa)	steel steel^d^ (MPa)
1		0	139	73	0.01	0.18	0.18	^e^	0.1 ± 0.0
2′	**11-Br**	2.96	120	33	1.5	4.6	4.88	0.8 ± 0.3^f^	3.6 ± 1.8
2″		4.78	104	17	0.04	1.8	1.81	3.4 ± 1.8^f^	1.8 ± 1.0
2‴		9.10	96	5	0.02	0.06	0.64	0.7 ± 0.4^f^	7.6 ± 3.7
3′	DEA	2.96	121	27	78	19	80.4	5.6 ± 0.8^g^	1.5 ± 0.9
3″		4.78	105	25	57	24	61.6	5.9 ± 0.7^g^	7.6 ± 3.1
3‴		9.10	99	5	29	15	33.1	5.5 ± 0.6^h^	6.1 ± 2.9
4′	EAE	2.96	119	30	74	55	92.2	5.8 ± 0.4^g^	6.7 ± 2.6
4″		4.78	106	21	58	47	74.2	6.1 ± 0.4^g^	15.2 ± 2.2
4‴		9.10	101	14	34	32	46.4	5.6 ± 0.6^h^	16.8 ± 0.9
5′	DEOA	2.96	116	29	190	82	203	3.4 ± 1.8^h^	9.1 ± 2.6
5″		4.78	101	11	110	48	119	6.0 ± 0.7^h^	15.3 ± 1.4
5‴		9.10	93	1	88	47	100	5.0 ± 0.3^h^	17.4 ± 1.6

a Determined by ^1^H NMR spectroscopy.

b Determined by DSC.

c ω = 1 rad s^–1^, measured at 180 °C.

d Determined by lap shear strength, mean adhesion ± one standard deviation reported.

e Measurement not possible due to lack of adhesion.

f Adhesive failure to metal.

g Stock break failure.

h Adhesive failure to plastic.

Fourier transform infrared (FT-IR) spectroscopy of the copolymers determined the presence of hydrogen bonding. A broad absorption band between 3600 and 3080 cm^–1^ can be attributed to the presence of O–H stretches between hydrogen-bonded hydroxyl units in all samples of **PP**_**EAE**_ and **PP**_**DEOA**_ (Figures 1 and S3);^41^ the observed peak broadness corresponds to the distribution of OH···O bond angles. Isolated hydroxyl groups were not observed, suggesting essentially complete participation in hydrogen-bonded networks. **PP**_**DEA**_ samples 3″ and 3‴ (Table 2), however, both display a broad peak at 3410 cm^–1^ (Figures 1 and S3), in the region of an aliphatic N···H hydrogen bonding environment despite the lack of H-bond donor groups.^45^ Given the apparent rheological network and adhesion results, *vide infra*, we attribute this to an exogenous hydrogen bond donor, likely to be aluminum hydroxide resulting from the hydrolysis of residual MAO despite purification by reprecipitation;^46^ an observed broad absorbance between 670 and 400 cm^–1^ may be attributed to these Al–O residues.^47^

**Figure 1:**
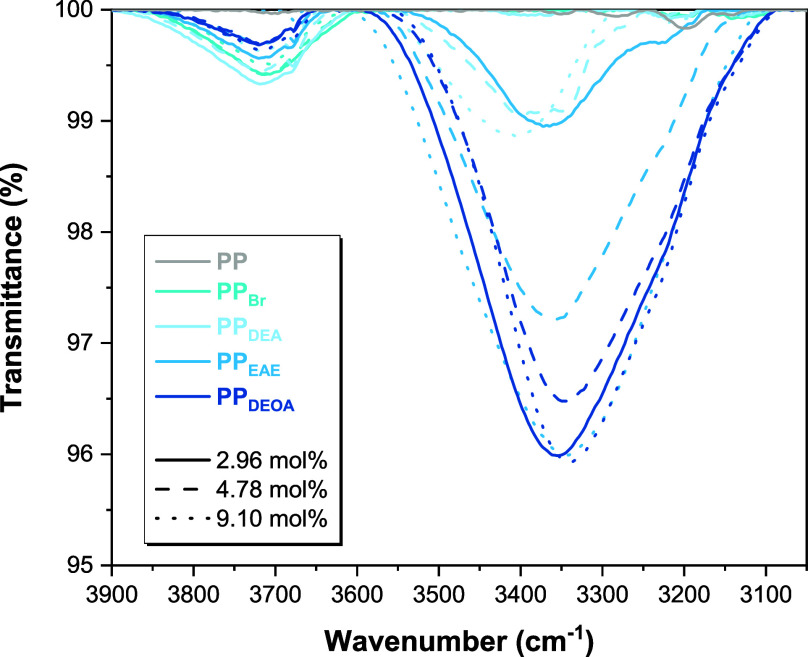
FT-IR spectrum of **PP**, **PP**_**Br**_, **PP**_**DEA**_, **PP**_**EAE**_, and **PP**_**DEOA**_ at 2.96, 4.78, and 9.10 mol % incorporation, shown between 3050 and 3900 cm^–1^.

### Rheological Evaluation of Functionalized Polypropylenes

The thermoresponsive viscoelastic behavior of PP is well-documented when accounting for *M*_n_ and crystallinity differences.^48^ It is important to note that direct comparisons between copolymers of different copolymer incorporation levels and their functionalized analogues are complex due to differing *M*_n_ and crystallinities, in addition to the variation in functionality. Recently, rheology and simulations have demonstrated the effects of hydroxyl-functionalized POs, with stable hydrogen bonding networks and nanophase hydroxyl aggregation expected in the polymer melt even at relatively low hydroxyl concentration.^41,49,200^

Small-amplitude oscillatory shear (SAOS) rheometry was conducted for all PP samples (Table 2 and Figures S4–S8) to evaluate the presence of a dynamic cross-linked network. Hydrogen bonding effects are expected to be more pronounced in the low shear regime, where the network reassociation time is faster than ω_c_^–1^ and hydrogen bonds are in dynamic equilibrium. Experiments were completed at 180 °C, the application temperature for subsequent lap shear strength testing, and in the frequency (ω) range 10^–2^–10^2^ rad s^–1^. **PP** and **PP**_**Br**_ behaved as expected with loss modulus (*G*″) greater than storage modulus (*G*′), indicative of a “liquid-like” viscosity-dominated regime, and crossover frequency, ω_c_ > 10^2^ rad s^–1^ (relaxation time τ < 0.01 s). With respect to increasing **11-Br** incorporation levels, **PP**_**Br**_ generally displayed increasing complex viscosity (η*) which may be attributed to increased branching density and comonomer-promoted entanglements as well as dipolar interactions.^50,51^

Evaluating *G*′ at a specific ω (1 rad s^–1^), for functional group conversions of the same parent polymers, it is possible to isolate the effects of changing heteroatom concentration and the impact of both hydrogen bond donor and acceptor moieties from molecular weight effects since the molecular weight distributions are identical (Figure 2). When accounting for each parent **PP**_**Br**_, each modification yields increasing *G*′: at 2.98 mol % incorporation, **PP**_**Br**_ = 1.53 kPa, **PP**_**DEA**_ = 78.1 kPa, **PP**_**EAE**_ = 74.2 kPa, and **PP**_**DEOA**_ = 186 kPa (Table 2). This observed increasing trend in *G*′ was likewise observed for the 4.78 and 9.10 mol % polymers. Increased *G*′ occurs due to restricted mobility and an enhancement to the polymer network in amine-modified polymers where hydrogen bonding is possible, through either intermolecular interactions or exogenous H-bond donors. By comparison with de-ashed polymers, prior studies have evidenced the impact of catalyst residues on rheological behaviors, with dynamic cross-links proposed to originate from hydrogen bonding and electrostatic interactions.^16^ Structural adhesives, especially ones able to undergo adhesion to low and high surface energy materials, typically require high *G*′ to resist deformation effectively and maintain a bond between structural components, enabling it to carry and distribute loads without significant creep or deformation.^52,53^ This is critical in applications like automotive, aerospace, and construction, where adhesives experience sustained forces over time.^54^

**Figure 2:**
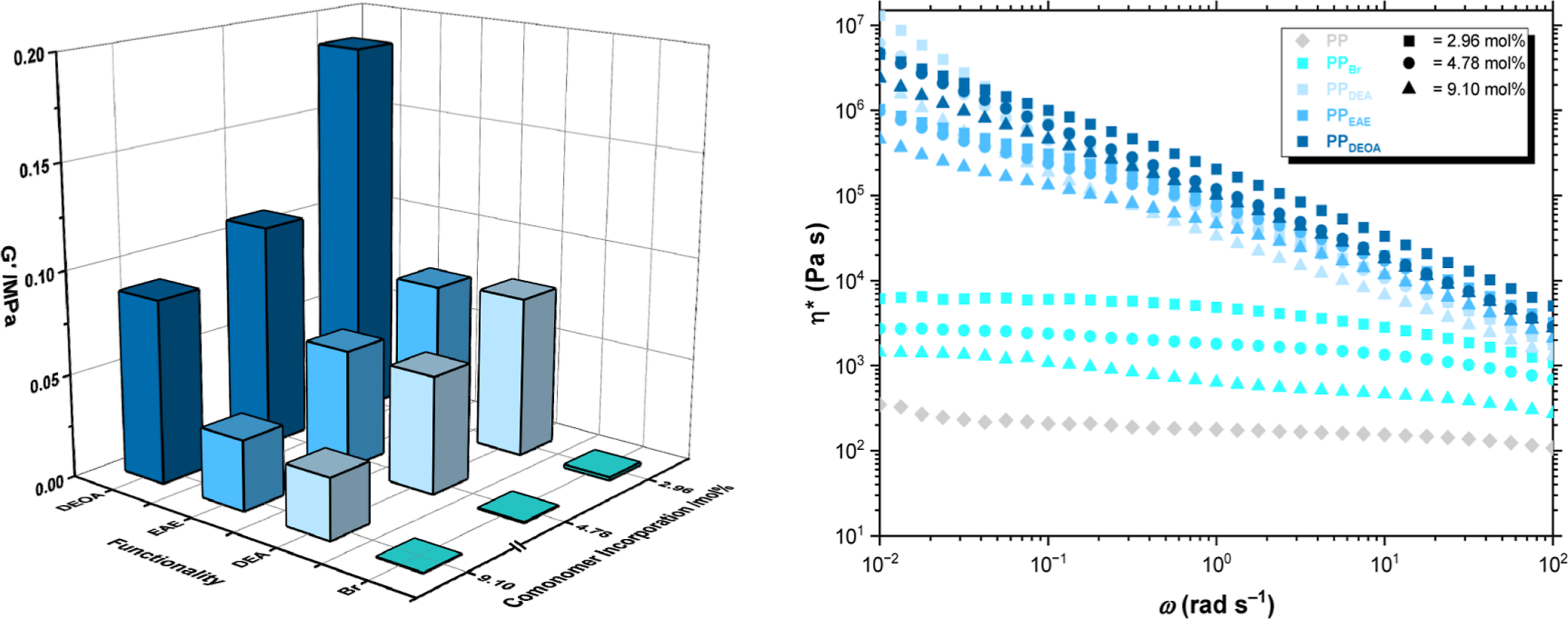
Small-amplitude oscillatory shear rheology of propylene copolymers measured at 180 °C. (Left) where *G*′ is reported at ω = 1 rad s^–1^ and related to comonomer incorporation and polymer functionality. (Right) complex viscosity, |η*|, as a function of oscillation frequency, ω.

All **PP**_**DEA**_ and **PP**_**DEOA**_ samples display an inverted relationship between *G*′ and *G*″ compared to **PP**_**Br**_ whereby *G*′ > *G*″, consistent with ω_c_ < 10^–2^ rad s^–1^. The longer relaxation times that this suggests (τ > 100 s) imply a “solid-like” material with a stable network that resists deformation over time even under relatively high shear. All **PP**_**EAE**_ samples evidence a modulus crossover (ω_c_; 4′ = 0.237 rad s^–1^, 4″ = 0.316 rad s^–1^, and 4‴ = 0.750 rad s^–1^; Figures S5–S7). Given lower ω_c_ can also suggest higher *M*_n_ or greater chain entanglement, both of which increase the resistance to flow and favor elastic behavior,^55–58^ in isolation, this data does not offer additional support for the presence of hydrogen bonding. However, a lower ω_c_ generally indicates a higher level of cross-linking or stronger interactions within the material as it maintains an elastic behavior even at lower frequencies. Interestingly, there is no observable crossover ω for all **PP**_**DEOA**_ samples as *G*′ > *G*″ consistent with ω_c_ < 10^–2^ rad s^–1^. Isolating the impact of *M*_n_ effects and taken in conjunction with observed **PP**_**EAE**_ data, we can infer that all **PP**_**DEOA**_ materials are dynamically cross-linked through hydrogen bonding to a greater extent than **PP**_**EAE**_ by virtue of the increased hydroxyl concentration, and both show orders of magnitude greater interactions than in the parent **PP**_**Br**_ polymers. The increased melt elasticity of these amine-functionalized PPs may in part be attributable to supramolecular networking effects mediated by intermolecular hydrogen bonding. In the case of **PP**_**DEA**_, the presence of exogeneous H-bond donors, observed by FT-IR spectroscopy, may be responsible for the observed network effect; alternatively polar-induced nanophase aggregates may act as supramolecular cross-links to produce a transient network.^41,49^

The strongest evidence for the existence of supramolecular hydrogen bonding networks arises from shear-thinning phenomena. At low shear rates, the network maintains equilibrium (the association time is less than ω^–1^ and hydrogen bonds quickly reform) and viscosity equals the zero-shear value (η = η_0_); shear thinning is attributable to the disruption of the equilibrium network which decreases viscosity. The shear sensitivity, measured as the slope of the shear-thinning region of η/η_0_ as a function of the shear rate, is therefore indicative of the extent of supramolecular networking. This normalization also removes the effects of molecular weight on viscosity allowing for a more direct comparison between samples of varying comonomer incorporation.^41^ This analysis was applied to SAOS complex viscosity η*/η*_0_ as a function of ω and to steady-shear viscosity η/η_0_ as a function of γ̇ with the zero-shear viscosities modeled according to the Carreau equation (Figures S9–S11).^59^ It is clear that amine-functionalized PPs have significantly greater zero-shear viscosities and display more pronounced shear thinning, both of which are consistent with a dynamic supramolecular hydrogen-bonded network in the melt phase.^41^

### Hot-Melt Adhesive Performance of Functionalized Polypropylenes

Interfacial adhesion is commonly investigated by lap shear strength (LSS) testing.^60,61^ Recently-reported work by Duchateau et al. highlighted the application of propylene-based hydroxyl-functionalized terpolymers for use as hot-melt adhesives (HMAs).^16^ Furthermore, we recently reported the LSS of low molecular weight hydroxyl-functional PPs, **PP**_**EAE**_ and **PP**_**DEOA**_, compared against **PP**, **PP**_**Br**_, and **PP**_**DEA**_.^29^ However, due to the low *M*_n_, cohesive failure was the predominate failure mode for the conducted LSS tests between aluminum and steel substrates. As such, the synthesis and application of higher *M*_n_ congeners was targeted to evaluate more accurately the efficiency of the propylene-based copolymers as HMAs. An application temperature of 180 °C was selected for two reasons: (1) to maximize the obtained adhesive force^16^ and (2) to promote diffusion of molecular chains between sample copolymers and the plastic substrate,^27^ the plastic being an untreated commercially supplied PP sheet. To this end, testing was completed between steel–steel and steel–plastic substrates to evaluate adhesion to both high and low surface energy substrates, aiming to exemplify the copolymers as multisubstrate HMAs.

The general trend in initial LSS test results between steel sheets (Figure 3 and Table 2) was analogous to our previously reported work with low *M*_n_ polymeric HMAs,^29^ albeit displaying significantly stronger cohesive forces and each sample failing at the copolymer–steel interface. The measured LSS is therefore indicative of the adhesive strength of the polymers. Unfunctionalized **PP** was found to be a poor HMA recording a very low mean adhesive force (<0.1 MPa). **PP**_**Br**_ displayed mean adhesive strengths greater than anticipated, with mean values somewhat increasing with **11-Br** incorporation. On inspection of the steel substrate after adhesive failure, a tarnish is present on the surface that is only observed for **PP**_**Br**_ samples. We anticipate this is a result of the generation of corrosive bromine species formed under the high temperature and pressure hot-melt conditions, increasing the overall surface area at the interface by etching the steel.^62^ **PP**_**DEA**_ displayed adhesive strengths similar to **PP**_**Br**_ (1.5–7.6 MPa) with the sample with the lowest functional group incorporation clearly performing the worst. The improvement compared to that of **PP** may be attributed to the enhanced polarity afforded by the diethylamine functionality. An additional adhesive mechanism may be the result of an acid–base reaction between the amine and surface hydroxyl groups resulting in an electrostatic attraction, which has been observed recently with amine/epoxy adhesives.^63^ **PP**_**EAE**_ and **PP**_**DEOA**_ both performed well as HMAs, recording the largest mean adhesive forces as 16.8 and 17.4 MPa, respectively, **PP**_**DEOA**_ displaying a 252-fold increase vs **PP**. It might be reasonably inferred that lone-pair-containing moieties interact via electrostatic effects with oxides present on the surface of the steel, subsequently improving adhesion; similarly, the available polymer functionality is responsible for eliciting the observed supramolecular network by rheological evaluation. Current commercial one- and two-component structural adhesives from a range of epoxy, urethane, methacrylate, and acrylic chemistries offer adhesive strengths between 9.6 and 42.7 MPa (*x̅* = 22 MPa) by LSS for steel connections,^54^ with the mechanism of adhesion (e.g., mechanical interlock, electrostatic, chemical) being actively investigated.^17,27,34,63^ Within each sample subset, mean increased adhesive performance can be ostensibly attributed to increasing heteroatom availability within the polymer matrix: 4′ = 6.7 MPa, 4″ = 15.2 MPa, and 4‴ = 16.8 MPa; 5′ = 9.1 MPa, 5″ = 15.3 MPa, and 5‴ = 17.4 MPa (Table 2).

**Figure 3:**
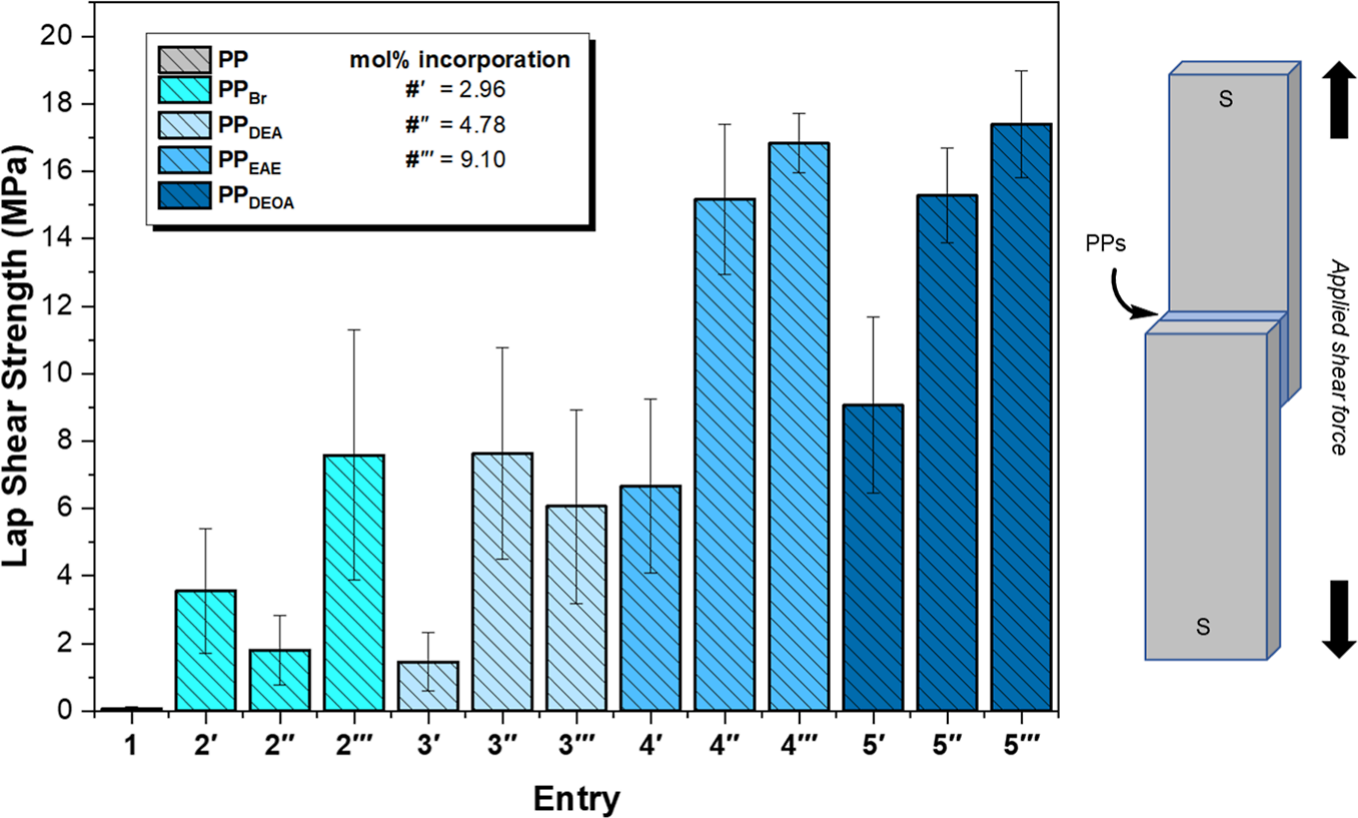
Lap shear strength tests with functional polymers as the adhesive interlayers between steel. Error bars represent standard deviation.

Functionalized POs offer a unique opportunity for MSA between plastic, through partial diffusion into the substrate forming adhesive–substrate chain entanglements, and steel through electrostatic interactions. This result is industrially and academically attractive, while the field remains underexplored due to the difficulties associated with synthesizing functionalized PPs in good yields. The LSS results between steel and plastic substrates are summarized in Table 2 and presented in Figures 4 and 5.

**Figure 4:**
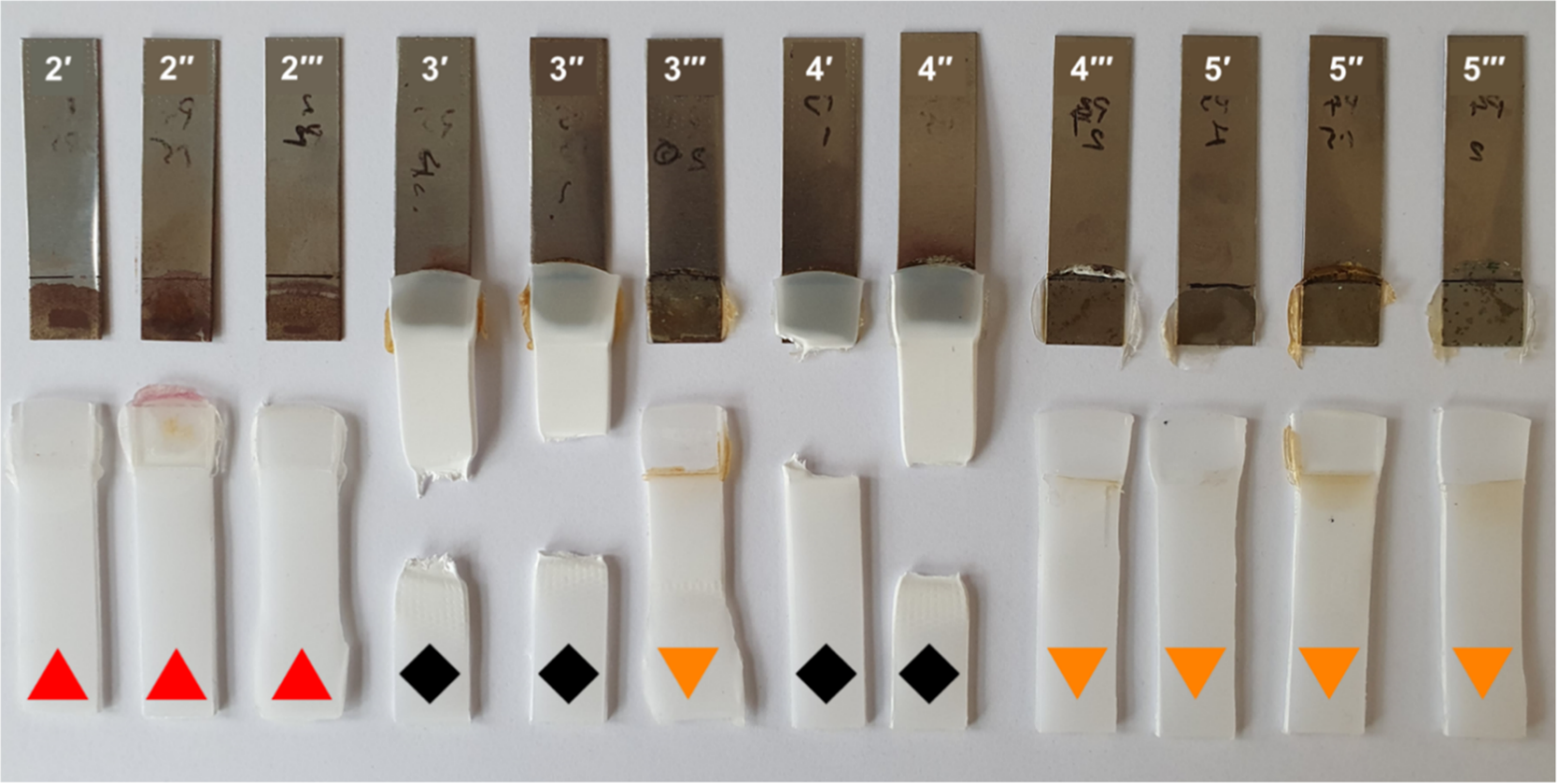
Lap shear strength samples evidencing failure modes: ▲ = adhesive (steel), ◆ = stock break, and ▼ = adhesive (plastic).

**Figure 5:**
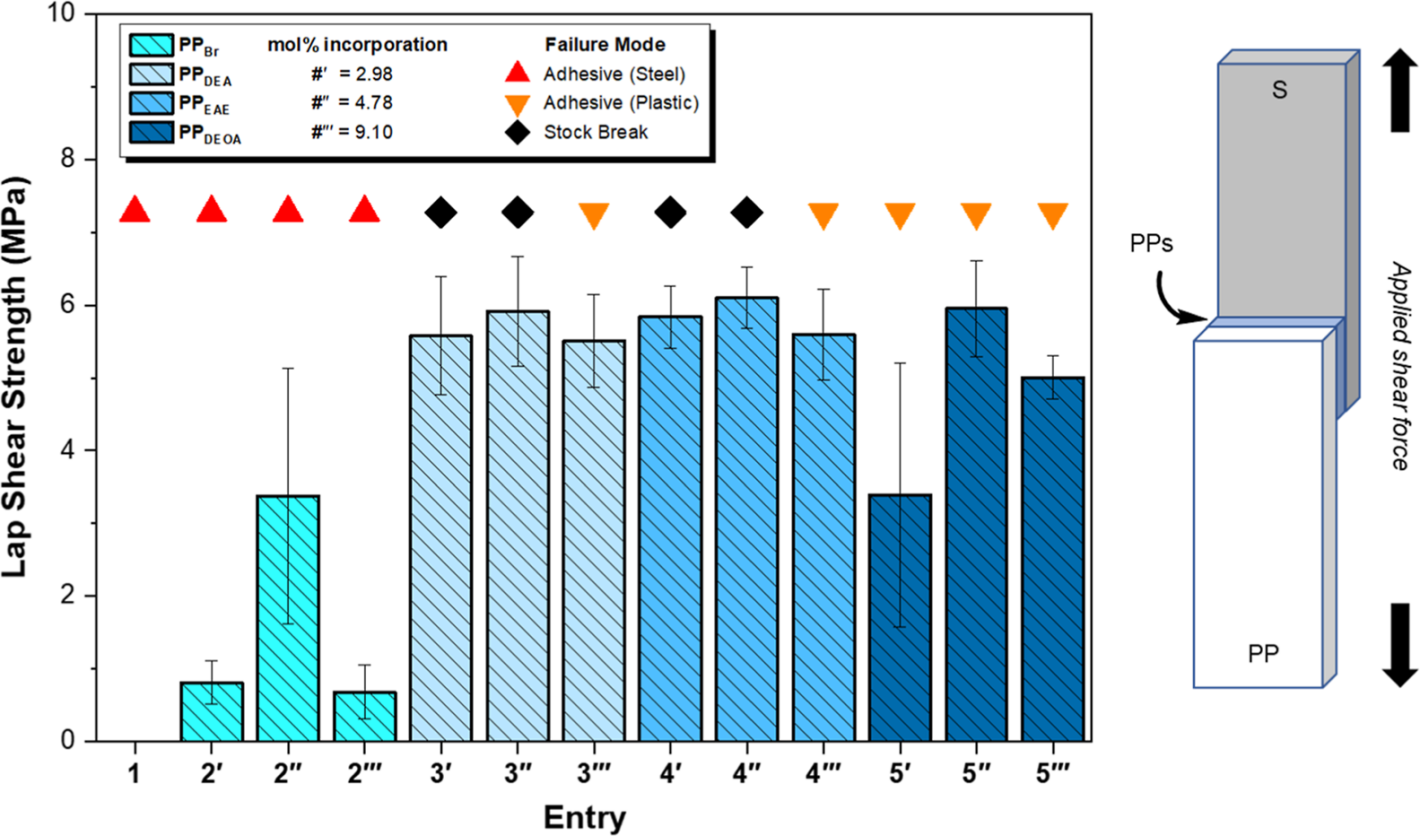
Lap shear strength tests with functional polymers as the adhesive interlayers between steel and commercial polypropylene. Error bars represent standard deviation.

Unsurprisingly, unfunctionalized **PP** was a poor HMA with no samples able to be tested due to a lack of adhesion to steel, preventing sample fabrication. **PP**_**Br**_ was likewise poor across the sample subset, with low adhesion to steel and the same tarnished steel indicative of corrosion. Interestingly, the failure mode, perhaps as expected, was adhesive failure to steel, with the **PP**_**Br**_ in all cases remaining adhered to the PP sheet. The observed failure mode, however, for **PP**_**DEA**_ (3′ and 3″) was stock break, whereby the PP sheet broke before the adhesive joint failed. This was not observed for 3‴; however, there was severe stress to the PP, evidenced by an increase in PP opacity, implying that the joint adhesion was only marginally weaker than that of the PP sheet under the experimental conditions. All but the greatest incorporation levels of **PP**_**EAE**_ likewise failed by stock break. The 9.10 mol %-incorporated **PP**_**EAE**_ and all **PP**_**DEOA**_ samples, however, underwent an alternate failure mode, this time adhesive failure to plastic due to decreased compatibility with the polypropylene substrate. As observed in the steel LSS results, the increase in heteroatom concentration correlates well with the increased adhesion to steel (Table 2). Variation in lap shear strength is attributed to inherent limitations of the standardized test method and minor differences in sample preparation, including adhesive overflow outside the bondline.^1,64^ Standardized measurements in pentuplicate aim to reduce the noise associated with stress concentration and crack propagation, which can occur outside of the interfacial zone.^65^

The mean adhesive force values for stock break failure modes are largely uninformative, instead being a proxy for the tensile strength of the PP sheet (though using the interface area rather than the substrate cross-section to calculate stress) and indicating that the adhesion strength is suitable for the substrate utilized. However, given the difference in failure modes across the series, it is likely that the selection of an optimal adhesive between steel and plastic can be obtained with the use of a plastic substrate with higher tensile strength. While failure mode evaluation is good evidence for our hypothesis, to further elucidate the structure of the interfacial monolayer and the mechanism of binding, special spectroscopic methods such as SFG could provide an alternate analytical approach.^66^ Furthermore, the trend can be qualitatively correlated to copolymer polarity, similar to that discussed in the steel–steel LSS results. There is a trade-off between increasing functionality to improve steel adhesion (also at the expense of *M*_n_ and crystallinity) and compatibility with the plastic substrate. Functionalized polymers with reduced polarity can form sufficient entanglements with the PP sheet and yet insufficiently adhere to metal, while those with the greatest degree of functionality are poorly miscible at the polymer interface yet retain significant adhesion to steel through the presence of heteroatoms. Therefore, the materials that failed by stock break, **PP**_**DEA**_ and **PP**_**EAE**_, sit in an optimal window regarding adhesive performance between both steel and plastic with the test specimens reaching the tensile strength limit of the PP substrate; importantly, the adhesive surpasses the requirements of the structural bond. The knowledge gained now allows further sets of functionalized PPs to be readily prepared to meet a diverse array of multisubstrate adhesive requirements. Further evaluation of the chemistry at the interfacial layer would be of interest and may prompt additional insight and development.

## Conclusion

We have reported the synthesis and characterization of a library of amine-modified polypropylene copolymers to assess effectively their adhesion to steel and commercial PP substrates. Rheological characterization evidenced viscoelastic phenomena believed to be the result of supramolecular hydrogen bonding. The increasing concentration of hydrogen bond acceptor and donor moieties is directly attributed to increases in the elasticity and shear sensitivity. The presence of functionality further contributed to adhesive strength, with hydroxyl functionality having a pronounced effect on LSS adhesion to steel. A maximum mean adhesive strength of 17.4 MPa is comparable to those of two-component epoxy resins. The dependency of adhesion between plastic and steel displayed an unexpected structure–functionality property relationship; observed failure modes transition from adhesive failure at the steel interface to stock break and finally adhesive failure at the plastic interface with increasing copolymer functionality. Tensile failure by stock break in this regime demonstrates that the adhesive joint strength is greater than the tensile strength of the substrate itself, rendering it wholly appropriate for adhesion to this material. This study successfully demonstrates the interplay between polymer polarity and binding ability to substrates of significantly different surface energies.

We have demonstrated that multisubstrate adhesion can be maximized by controlling the molecular weight and nature and amount of functionality of PP, balancing the requirements of forming entanglements to the PP substrate and binding to the polar metal substrate. The two-step copolymerization and postmodification platform utilized has already been shown to be capable of producing diverse functionalized PPs and can readily expand this class of hot-melt adhesives. We believe that this allows for the preparation of broad classes of HMAs, adaptable to various multisubstrate systems, applicable to many industrial adhesive applications where conventional systems are inadequate.

## Supplementary Material



## Notes

General details and instrumentation; polymer NMR and FT-IR spectroscopy and DSC and SEC characterization; polymer rheology; lap shear strength; modifier polarity assessment; and solution-phase copolymerization of propylene/**11-Br** by Zr (PDF)
