# Supplementary material for: Functionalized Polypropylene Copolymers as Multisubstrate Hot-Melt Adhesives
Source: ACS Appl Mater Interfaces. 2025 Jun 3;17(23):34592–601. doi: 10.1021/acsami.5c07594 (PMC12163929; doi:10.1021/acsami.5c07594)
Supplement: Supplementary file 1 [file am5c07594_si_001.pdf]

Electronic Supporting Information

Functionalized polypropylene copolymers as multi-substrate hot melt adhesives

Alexander Evans, Clement G. Collins Rice, Zoë R. Turner, and Dermot O'Hare*

Chemistry Research Laboratory, Department of Chemistry, University of Oxford, 12 Mansfield Road, OX1 3TA Oxford, UK.
E-mail: dermot.ohare@chem.ox.ac.uk

Contents

1. **General details and instrumentation** S2
2. **Polymer NMR and FT-IR spectroscopy, DSC and SEC characterisation** S3
3. **Polymer rheology** S5
4. **Lap shear strength** S9
5. **Modifier polarity assessment** S10
6. **Solution phase copolymerization of Propylene/11-Br by Zr** S11
7. **References** S13

1. General details and instrumentation

General procedures. Air- and moisture-sensitive compounds were manipulated under an inert atmosphere of nitrogen, using standard Schlenk line techniques[1] on a dual manifold vacuum/nitrogen line or in an MBraun Labmaster 100 glovebox.

Hexanes and toluene were dried using an MBraun SPS 800 solvent purification system, stored over a potassium mirror, and degassed under partial vacuum before use.

Solution NMR spectroscopy. NMR spectra were recorded on a Bruker AVD 500 MHz. Spectra were recorded at 393 K and referenced internally to the residual *protio* solvent resonance ($C_2D_2Cl_4$). Chemical shifts, δ, are reported in parts per million (ppm) relative to tetramethylsilane (δ = 0 ppm). Convection compensated diffusion experiment was carried out using Bruker pulse program "dstebpgp3s" on a Bruker AVD 500 MHz, $C_2D_2Cl_4$, 393 K.

Gel permeation chromatography. Gel permeation chromatography (GPC) was performed by Ms Liv Thobru, Ms Sara Rund Herum, and Ms Rita Jenssen (Norner AS, Norway) on a high temperature gel permeation chromatograph with an IR5 infrared detector (GPC-IR5). Samples were prepared by dissolution in 1,2,4-trichlorobenzene (TCB) containing 300 ppm of 3,5-di-*tert*-buty-4-hydroxytoluene (BHT) at 160 °C for 90 minutes and then filtered with a 10 µm SS filter before being passed through the GPC column. The samples were run under a flow rate of 0.5 mL min^{-1} using TCB containing 300 ppm of BHT as mobile phase with 1 mg mL^{-1} BHT added as a flow rate marker. The GPC column and detector temperature were set at 145 and 160 °C respectively.

Differential scanning calorimetry. Differential scanning calorimetry was performed on a Perkin Elmer DSC 4000 System within a temperature range of 30–200 °C at a rate of 5 K min^{-1}. Polymer samples were sealed in 50 µL aluminium crucibles. An empty crucible was used as a reference, and the DSC was calibrated using indium and zinc.

2. Polymer NMR and FT-IR spectroscopy, DSC and SEC characterisation

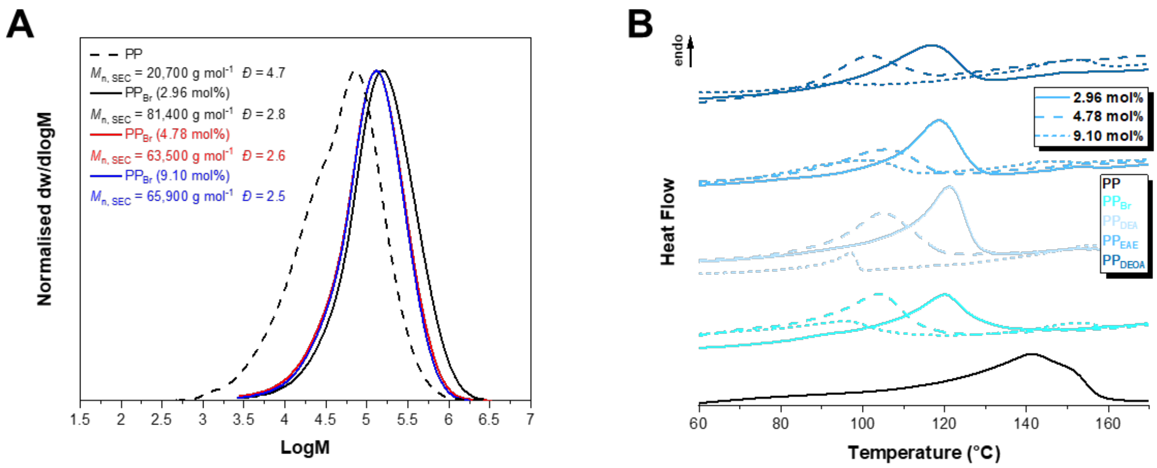

Figure S1 (**A**) SEC traces of **PP** and **PP$_{Br}$** at 2.96, 4.78, and 9.10 mol% incorporation. (**B**) DSC traces **PP**, **PP$_{Br}$**, **PP$_{DEA}$**, **PP$_{EAE}$**, and **PP$_{DEOA}$** at 2.96, 4.78, and 9.10 mol% incorporation. Crystallization temperatures (T_c) by DSC are reported as follows: **1** 99.2 °C, **2′** 86.7 °C, **2″** 67.4 °C, **2‴** 68.0 °C, **3′** 89.4 °C, **3″** 59.5 °C, **3‴** 36.8 °C, **4′** 78.9 °C, **4″** 33.2 °C, **4‴** < 30 °C, **5′** 63.3 °C, **5″** 33.9 °C, **5‴** < 30 °C (Table 2).

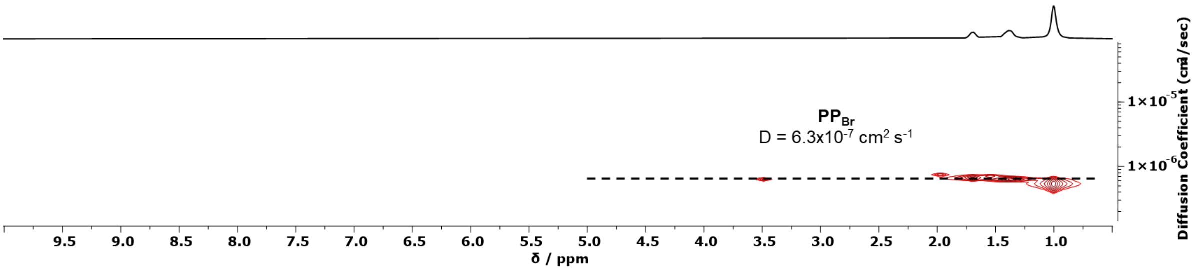

Figure S2 DOSY ^{1}H NMR spectra (500 MHz, $C_2D_2Cl_4$, 393 K) of **PP$_{Br}$**.

For samples **PP$_{DEA}$**, **PP$_{EAE}$**, and **PP$_{DEOA}$** diffusion experiments were carried out with convection compensation, however due to the rapid relaxation of nitrogen adjacent protons –NC***H*$_2$**R– it was impossible to measure accurate diffusion coefficients and evidence coupling by new characterisation methods. In previously reported work, resonances in ^{1}H NMR spectra appeared broad and this observation was potentially explained by an increase in the relaxation rate caused by the adjacent quadrupolar ^{14}N. A lack of HMBC cross-peaks whilst still evidencing HSQC cross-peaks supported this. Additionally, when carrying out the diffusion experiment the relaxation times were fast (< ~20 ms) as observed in the T_1 inversion recovery experiment ran prior to the diffusion experiment. Previously, experimentally HSQC equivalent delays were optimised for $^1J_{CH}$ 145 Hz coupling (3 ms delay) whereas HMBC experiments were optimised for a $^3J_{CH}$ 8 Hz (125 ms delay), giving the nuclei sufficient time to relax and preventing the appearance of any cross-peak to carbons adjacent to nitrogen, consequently this new evidence supports this hypothesis.[2-4]

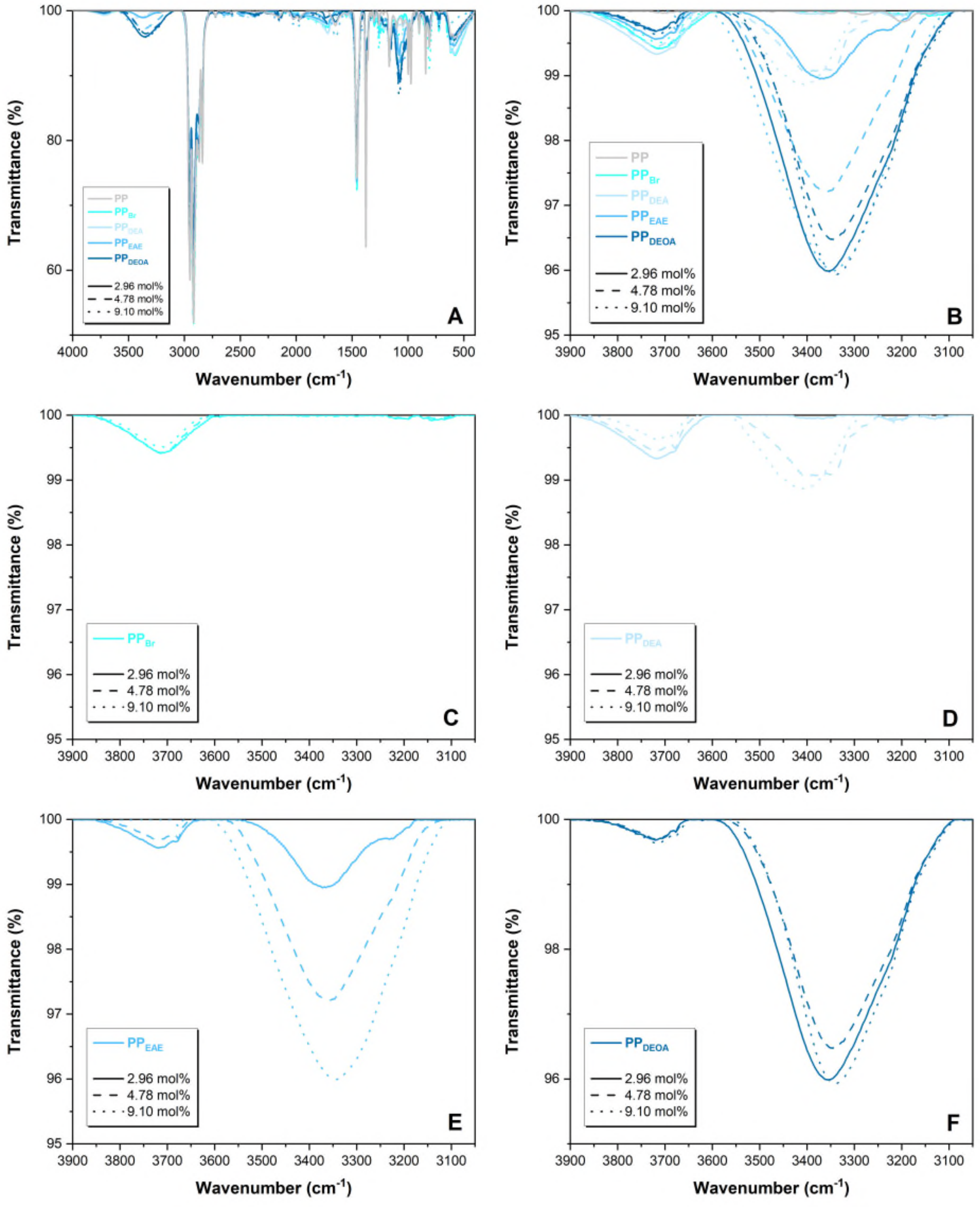

Figure S3 FT-IR spectrum of (**A**) **PP**, **PP$_{Br}$**, **PP$_{DEA}$**, **PP$_{EAE}$**, and **PP$_{DEOA}$** at 2.96, 4.78, and 9.10 mol% incorporation; (**B-F**) shown between 3050-3900 cm^{-1} of **PP$_{Br}$**, **PP$_{DEA}$**, **PP$_{EAE}$**, and **PP$_{DEOA}$** at 2.96, 4.78, and 9.10 mol% incorporation.

3. Polymer rheology

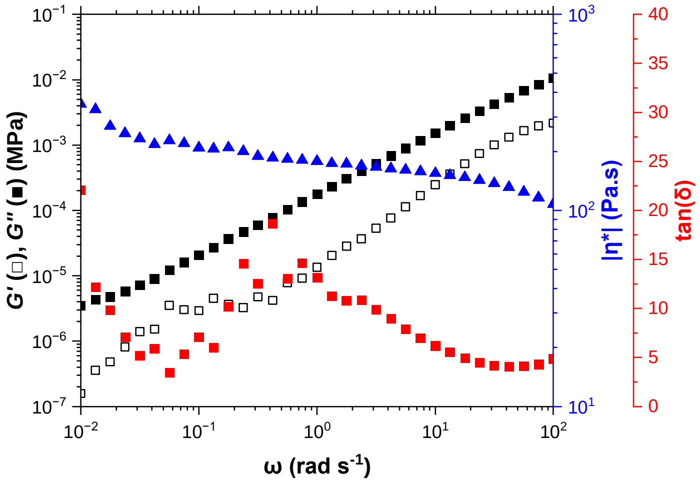

Figure S4 Frequency sweep rheometric curve measured at 180 °C for **PP**. Storage modulus, G′; loss modulus, G″; complex viscosity, |η*|; phase angle, δ; angular frequency, ω.

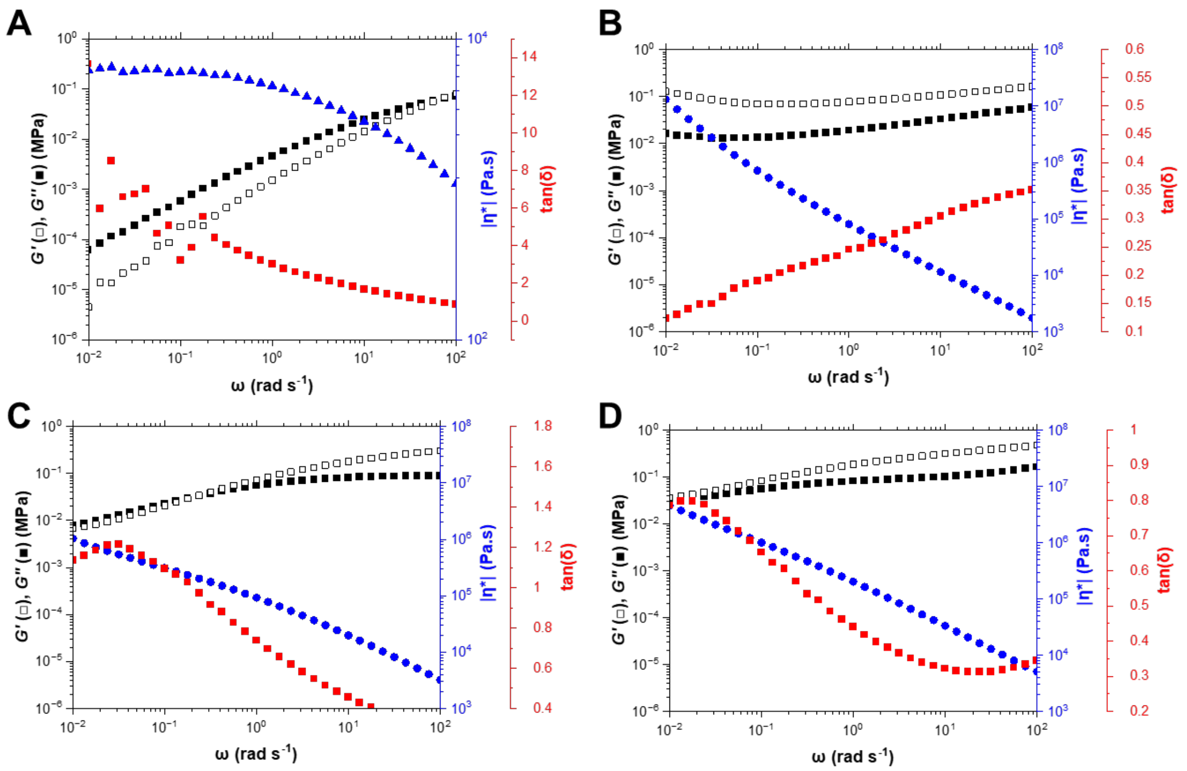

Figure S5 Frequency sweep rheometric curve measured at 180 °C for **(A) PP$_{Br}$**, **(B) PP$_{DEA}$**, **(C) PP$_{EAE}$**, and **(D) PP$_{DEOA}$**; 2.98 mol% incorporation. Storage modulus, G′; loss modulus, G″; complex viscosity, |η*|; phase angle, δ; angular frequency, ω.

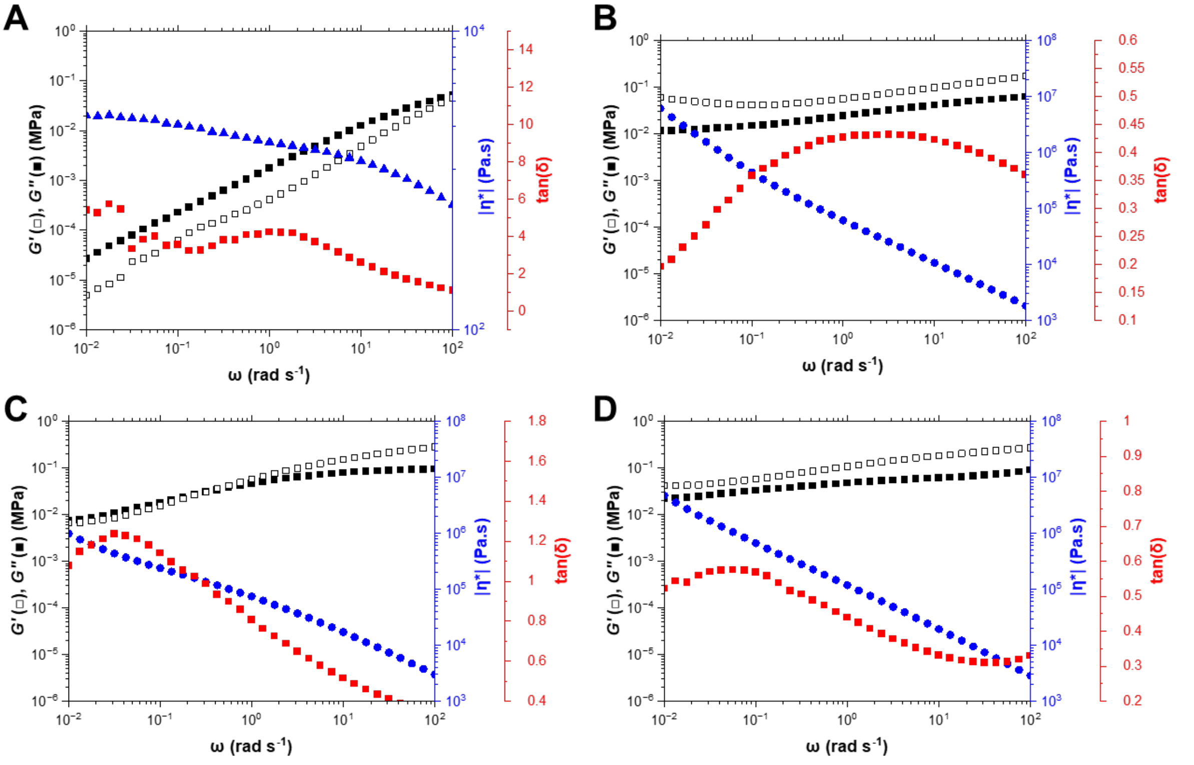

Figure S6 Frequency sweep rheometric curve measured at 180 °C for (**A**) **PP**$_{\mathbf{Br}}$, (**B**) **PP**$_{\mathbf{DEA}}$, (**C**) **PP**$_{\mathbf{EAE}}$, and (**D**) **PP**$_{\mathbf{DEOA}}$; 4.78 mol% incorporation. Storage modulus, G′; loss modulus, G″; complex viscosity, |η*|; phase angle, δ; angular frequency, ω.

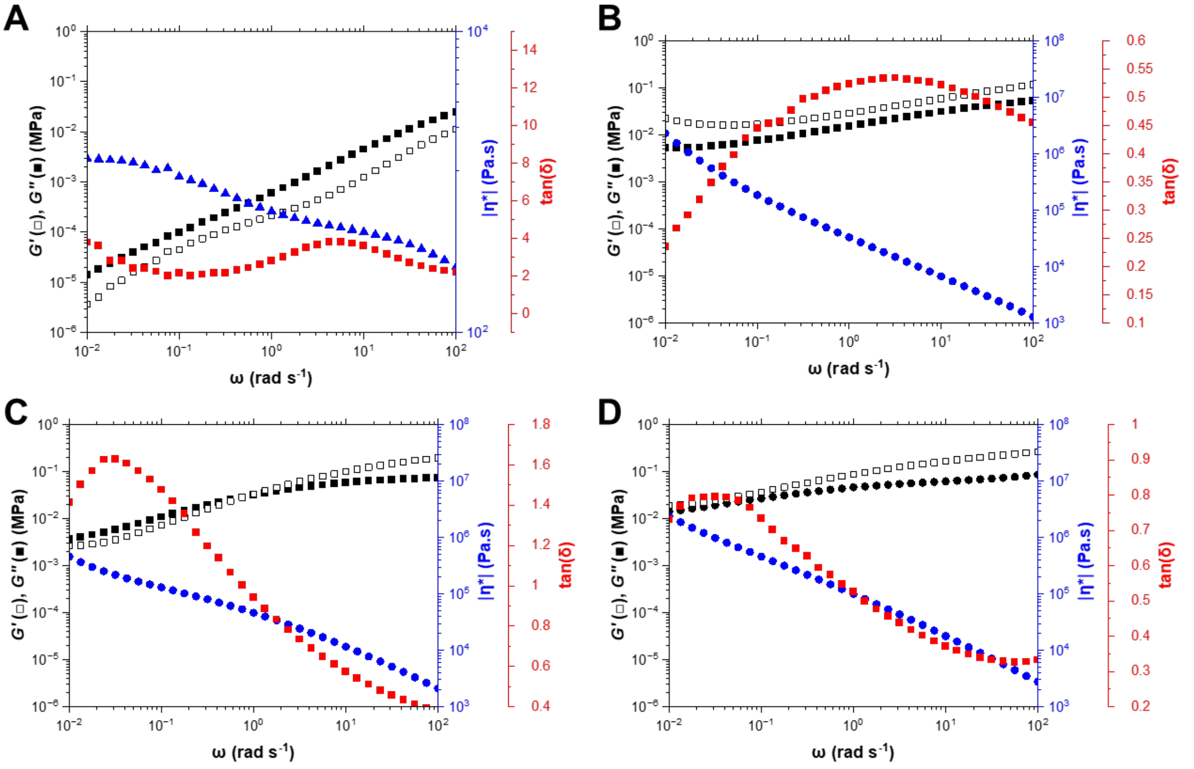

Figure S7 Frequency sweep small-amplitude oscillatory shear rheometry measured at 180 °C for (**A**) **PP**$_{\mathbf{Br}}$, (**B**) **PP**$_{\mathbf{DEA}}$, (**C**) **PP**$_{\mathbf{EAE}}$, and (**D**) **PP**$_{\mathbf{DEOA}}$; 9.10 mol% incorporation. Storage modulus, G′; loss modulus, G″; complex viscosity, |η*|; phase angle, δ; angular frequency, ω.

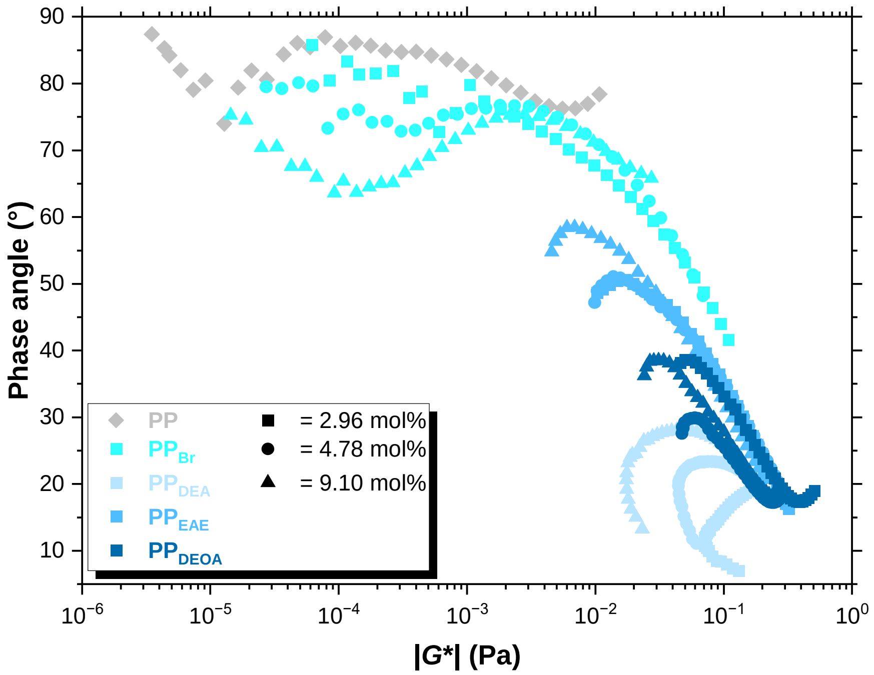

Figure S8 van Gurp-Palmen plot (phase angle as a function of the complex modulus), for propylene (co)polymers. A phase angle (δ) approaching 90 ° at low G^* indicates a material response dominated by viscous flow (**PP** and **PP$_{\mathbf{Br}}$**), while decreasing δ with decreasing G^* is indicative of increased elasticity.[5] The enhanced elasticity of amine-functionalized PP can be rationalized through supramolecular network effects acting as physical crosslinks.

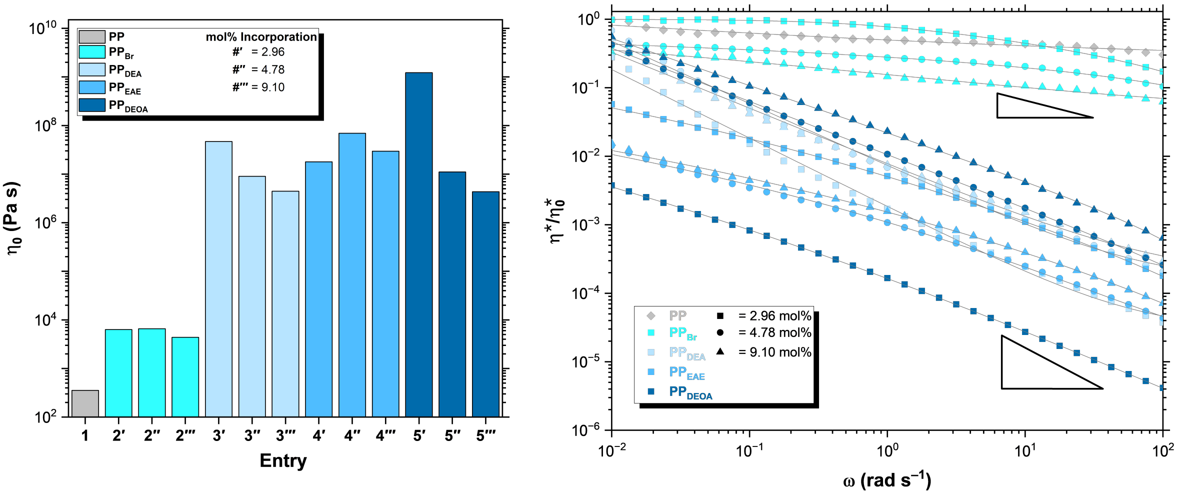

Figure S9 (Left) Zero-shear viscosity obtained from SAOS by fitting complex viscosity according to the Carreau equation, $\boldsymbol{\eta}^*(\boldsymbol{\omega}) = \boldsymbol{\eta}_0^*\left[\mathbf{1} + (\boldsymbol{\lambda\omega})^2\right]^{\left(\frac{\boldsymbol{n}-\mathbf{1}}{\mathbf{2}}\right)}$. (Right) Complex viscosity normalized by η^*_0 as a function of oscillation frequency. According to the Cox-Merz rule,[6] there is a direct correspondence between $\boldsymbol{\eta}^*(\boldsymbol{\omega}) \Leftrightarrow \boldsymbol{\eta}(\dot{\boldsymbol{\gamma}})$ though this generally holds more strongly for therorheologically simple materials, it has been demonstrated for virtually all single-phase polymer melts.[7, 8]

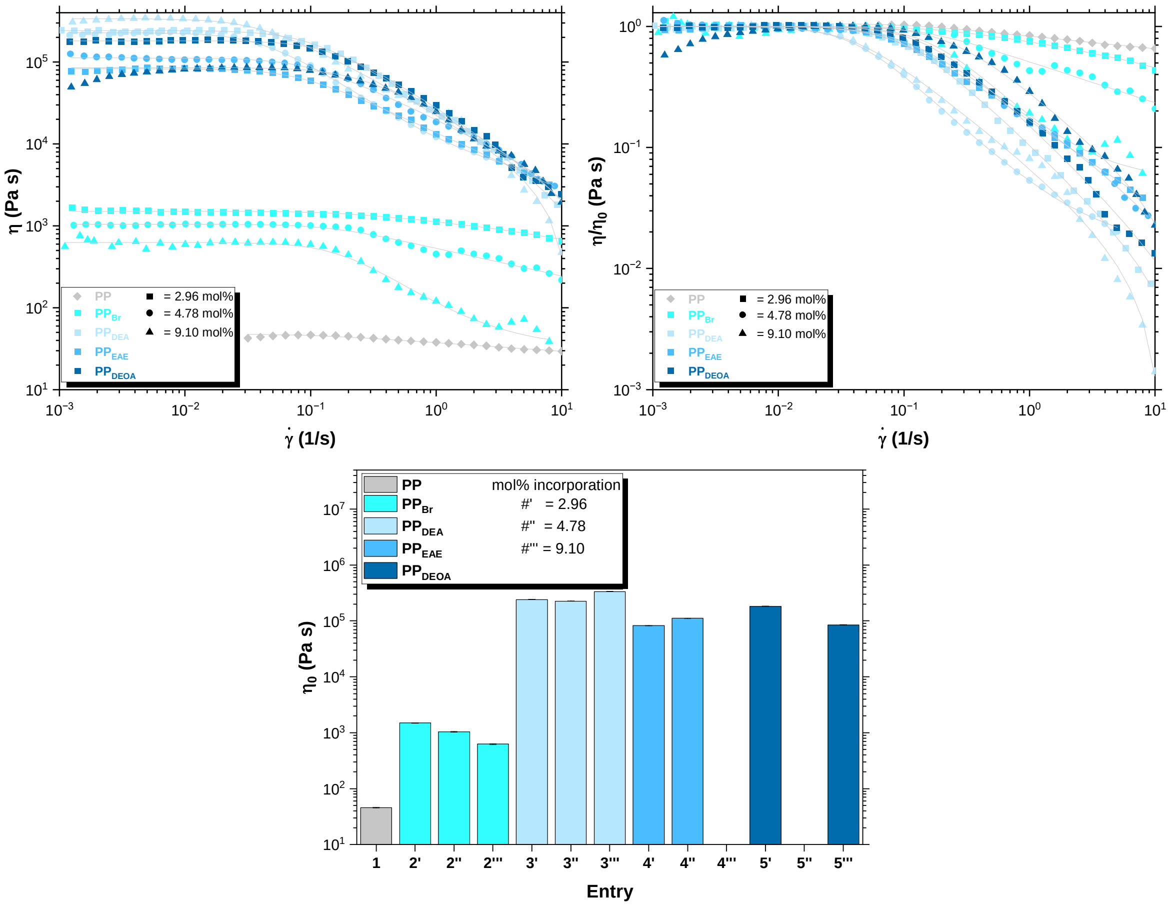

Figure S10 Steady-shear rheometry of **PP**, **PP$_{Br}$**, **PP$_{DEA}$**, **PP$_{EAE}$**, and **PP$_{DEOA}$**. Samples were prepared as for SAOS, and the rheological response was measured at 180 °C across a logarithmic sweep of shear rates from 10^{-3} to 10^{2} s^{-1}. (Top left) Viscosity, η, as a function of strain rate $\dot{\gamma}$ fitted according to the Carreau equation, $\boldsymbol{\eta(\dot{\gamma}) = \eta_0\left[1+(\lambda\dot{\gamma})^2\right]^{\left(\frac{n-1}{2}\right)}}$. (Top right) Viscosity normalized by the zero-shear viscosity. (Bottom) Zero-shear viscosity obtained from the Carreau equation. Normalization removes the effects of molecular weight and distribution with the shear sensitivity of the supramolecular network visible as shear thinning phenomena at high shear rate. At low $\dot{\gamma}$, the hydrogen bonds are quickly reformed and $\eta = \eta_0$, but at shear rates where the association time is slower than $\dot{\gamma}^{-1}$ the equilibrium between isolated and networked hydroxyls favours isolation and the measured viscosity decreases. The slope of the shear thinning therefore corresponds to the extent of the supramolecular network.

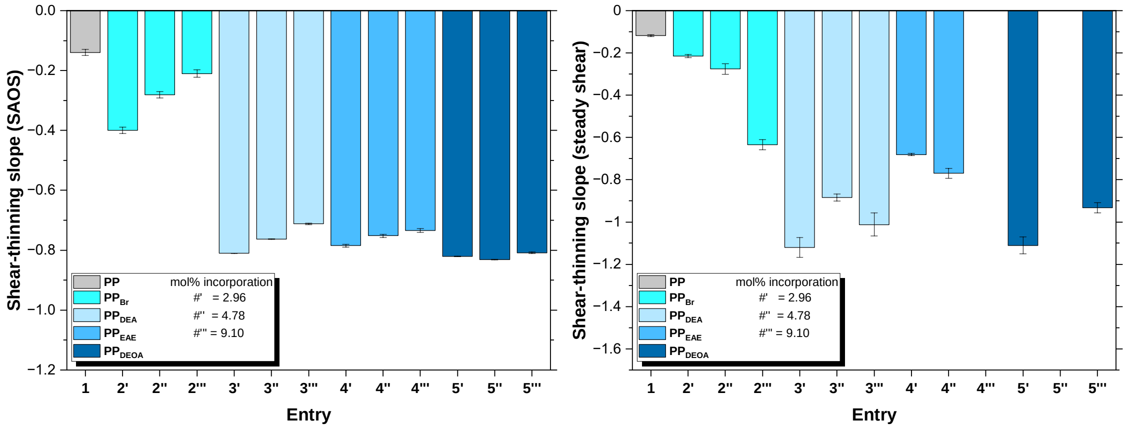

Figure S11 Shear-thinning slope obtained from (left) SAOS and (right) steady-shear rheometry, normalized according to the zero-shear viscosity and fitted linearly in the high-shear non-Newtonian region.

4. Lap shear strength

Table S1 Lap shear strength measured adhesive forces

Entry	Modifier	Steel-Steel (MPa)	σ	Steel-Plastic (MPa)	σ
**1**		0.0691	0.0487	0.000	
**2′**	Br	3.555	1.835	0.811	0.297
**2″**		1.808	1.028	3.379	1.754
**2‴**		7.585	3.718	0.677	0.370
**3′**	DEA	1.458	0.866	5.579	0.810
**3″**		7.638	3.139	5.916	0.749
**3‴**		6.064	2.872	5.508	0.639
**4′**	EAE	9.061	2.611	5.837	0.426
**4″**		15.166	2.239	6.098	0.421
**4‴**		16.831	0.877	5.590	0.624
**5′**	DEOA	6.663	2.581	3.392	1.816
**5″**		15.277	1.412	5.951	0.665
**5‴**		17.395	1.574	5.005	0.295

Determined by lap shear strength, mean adhesion and standard deviation reported ($N \geq 4$).

5. Modifier polarity assessment

A somewhat crude quantitative assessment of the effect of polarity, whereby incorporation is multiplied by a normalised coefficient related to any of modifier boiling point, electric dipole moment, summed heteroatom electronegativity or lone pairs, correlates fairly well to increasing adhesive strength. Importantly, this makes many assumptions and ignores a variety of intrinsic variables and should be interpretated generally and qualitatively with respect to the trends in adhesive forces.

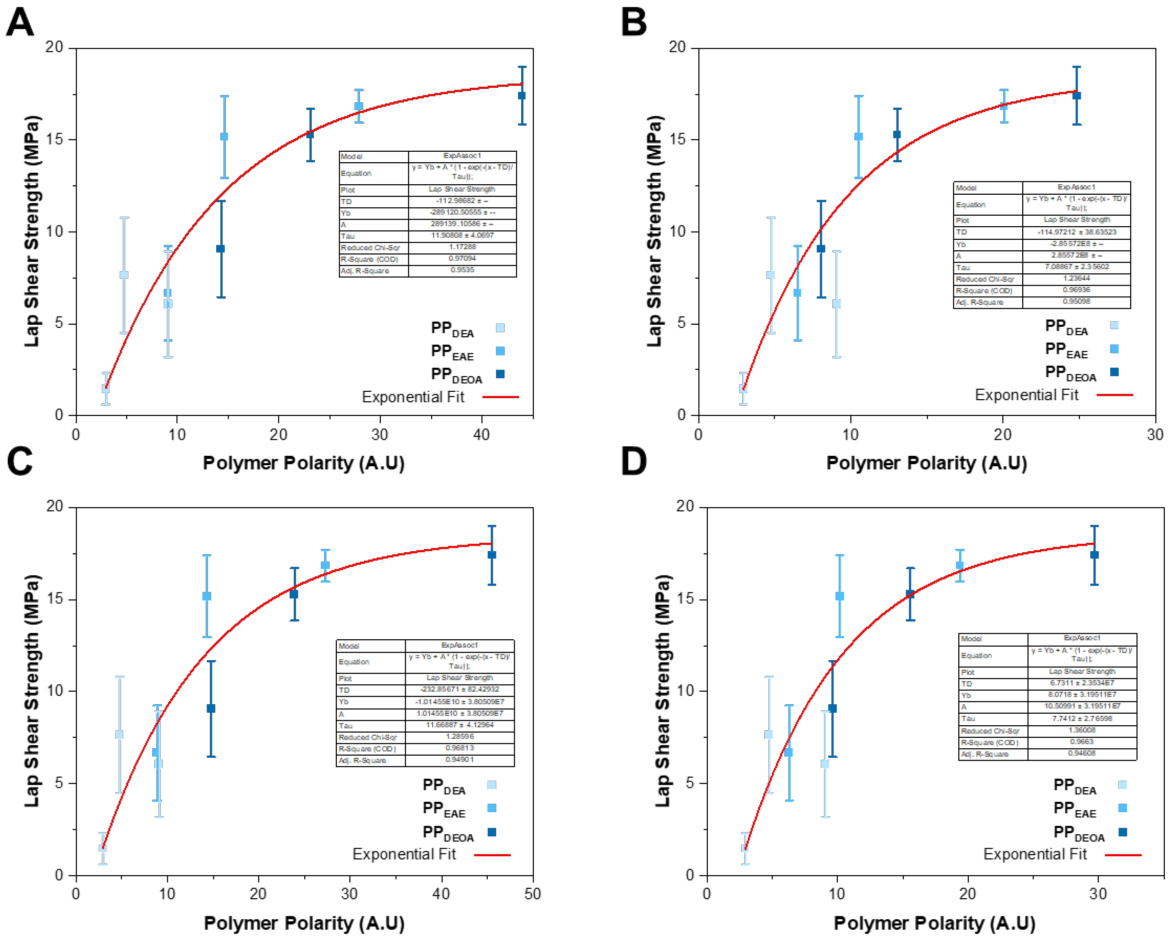

Figure S12 Evaluation of the relationship between lap shear strength and modifier polarity, where modifier is normalised from a physical constant: (**A**) modifier boiling point, (**B**) modifier electric dipole moment, (**C**) modifier summed lone pairs, and (**D**) modifier sum of heteroatom electronegativity.

6. Solution phase copolymerization of Propylene/11-Br by Zr

Further investigations into the solution-phase copolymerization of **Zr** were undertaken, establishing the tunability and affinity for **11-Br** incorporation in addition to a Fineman-Ross analysis.

Table S2 (Co)polymerization of propylene (and 11-bromo-1-undecene).[a]

Entry	[**11-Br**] (mM)	Activity[b]	Yield (g)	Incorporation (mol%)[c]	M_w (kg mol^{-1})[d]	$Đ_M$[d]	T_m[e] (°C)	Crystallinity[e] (%)
**1**	45.1	6984 (782)	5.6	1.48	216.6	4.49	136	43
**2**	89.4	3422 (464)	4.1	3.07	232.0	4.2	115	51
**3**	132.8	2119 (322)	2.5	4.80	173.0	2.9	99	25
**4**	175.3	989 (44)	1.2	6.37	155.0	2.9	89	17
**5**	217.1	643 (44)	0.8	7.25	149.6	3.0	83	28
**6**	258.0	559 (141)	0.7	8.64	119.2	3.3	77	16

[a]Conditions: dimethylsilylene bis(2–methyl–4–phenyl–1–indenyl) zirconium dichloride (**Zr**), MAO used as a cocatalyst and scavenger with $[Al]_0$:[**Zr**]$_0$ = 1000:1. Molar ratio of $[TIBA]_0$:[**11-Br**]$_0$ = 1:10, 0.5 mL toluene, 49.5 mL hexanes, propylene 2 bar, polymerization temperature = 50 °C, 0.5 h. Polymerization were performed at least in duplicate and mean values reported, with one standard deviation reported in brackets (σ). [b]$kg_{PP}\ mol_{Zr}^{-1}\ h^{-1}\ bar^{-1}$. [c]Determined by ^{1}H NMR. [d]Determined by SEC using the Mark-Howink correction for PP. [e]Determined by DSC.

Evaluation of the copolymerization between propylene and **11-Br** has been attempted for this system using data reported in **Table S2** and summarised below in **Table S3** to yield estimated reactivity ratios for propylene (r_P = 3.47 ± 0.08) and **11-Br** (r_B = 0.83 ± 0.25) (Figure S13). Using the Fineman-Ross method,[9] and taking the approximate concentration of propylene in hexanes according to Kissin's equation, $[P] \approx p \cdot 0.00272 \cdot exp\left(\frac{3260}{1.98 \cdot T}\right)$ for partial pressure, p, in bar and absolute temperature T,[10] it is possible to estimate the reactivity ratios.

$$f = F\frac{r_1F + 1}{r_2 + F} \Rightarrow \frac{F}{f}(f - 1) = r_1\frac{F^2}{f} - r_2$$
$$r_1 = \frac{k_{11}}{k_{12}};\ r_2 = \frac{k_{22}}{k_{21}}$$

where F = [P]/[**11-Br**] in feed, f = [P]/[**11-Br**] in copolymer, and k_{nm} is the rate constant for the insertion of monomer m after monomer n.

Table S3 Fineman-Ross data for P/**11-Br** copolymerisation. F = [P]/[**11-Br**] in feed, f = [P]/[**11-Br**] ($= \frac{1}{x_{11-Br}} - 1$) in copolymer. [P] calculated from Kissin's equation, for partial pressure, *p*, in bar and absolute temperature *T*: $[P] \approx p \cdot 0.00272 \cdot exp\left(\frac{3260}{1.98 \cdot T}\right)$.[10] [a]Polymerization conditions: **M2**, MAO used as a cocatalyst and scavenger with $[Al]_0$:$[\mathbf{M2}]_0$ = 1000:1. Molar ratio of $[TIBA]_0$:$[\mathbf{11\text{-}Br}]_0$ = 1:10, 0.75 mL toluene, 49.25 mL hexanes, propylene 2 bar. Polymerization and analysis were performed in duplicate and mean values are reported.

**11-Br** /μL	T/°C	[P] /mM	[**11-Br**] /mM	$x_{11\text{-}Br}$ (mol%)	F	f
500	50	888	45	1.48	5.73	19.21
1000	50	888	91	3.07	3.02	9.45
1500	50	888	137	4.80	2.13	6.18
2000	50	888	182	6.37	1.62	4.55
2500	50	888	228	7.25	1.19	3.60
3000	50	888	273	8.64	1.00	2.94

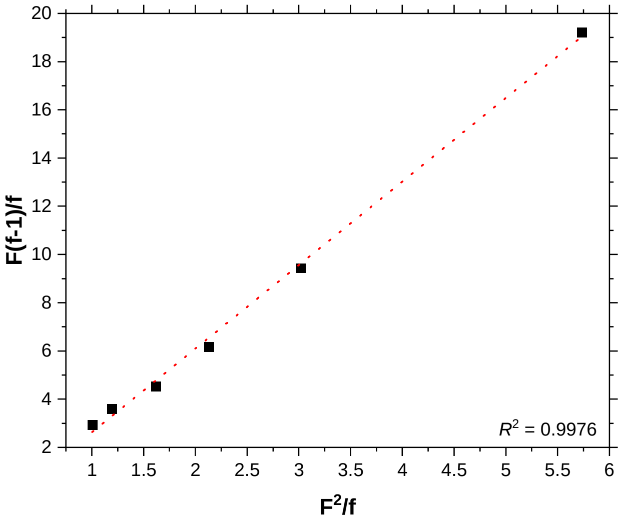

Figure S13 Fineman-Ross plot for propylene/**11-Br** copolymerisation at T_p = 50 °C and [**11-Br**] = 45.5–273.0 mM.

In the literature, reactivity ratios pertaining to ethylene/propylene copolymerizations have been well summarised[11] and extended for 1-hexene copolymerizations utilising three Kaminsky-Sinn-type catalysts.[12] The product of the reactivity ratios may be illustrated for a propylene (P), **11-Br** (B) copolymer as follows:

$r_P \bullet r_B = 1$	random	PPBPBPPBPBBP
$r_P \bullet r_B < 1$	alternating	PBPBPBPBPBPB
$r_P \bullet r_B > 1$	block	PPPPBPBBBBPP

Given $r_P \bullet r_B = 2.89 \pm 0.87$, evidence suggests block like copolymers are the product of copolymerization. Given $r_B \approx 1$, when accounting for standard error in the linear regression, we might reasonably expect **11-Br** to be substantially isolated within propylene segments. $^{13}C\{^1H\}$ NMR spectroscopy data supports this conclusion with a single resonance (45.82 ppm) for methine carbons at the copolymer junction consistent with isolated comonomers afforded by a single insertion of **11-Br** followed by a PP block in line with data previously reported for *rac*-ethylenebis(indenyl) zirconium dichloride (**EBI**).[4] Comparatively, the propensity for comonomer insertion is lower *vs* **EBI** (r_B = 1.36 ± 0.14); possibly explained by the greater steric constraints imposed by the SPALEK ligand towards monomer insertion.

7. References

(1) Shriver, D. F.; Drezdon, M. A. *The Manipulation of Air-Sensitive Compounds*; Wiley, 1986.

(2) Wong, T. C.; Collazo, L. R.; Guziec, F. S. 14N and 15N NMR studies of highly sterically hindered tertiary amines. *Tetrahedron* **1995**, *51* (3), 649-656.

(3) Jarvis, J. A.; Haies, I. M.; Williamson, P. T.; Carravetta, M. An efficient NMR method for the characterisation of 14N sites through indirect 13C detection. *Phys. Chem. Chem. Phys.* **2013**, *15* (20), 7613-7620.

(4) Evans, A.; Casale, O.; Morris, L. J.; Turner, Z. R.; O'Hare, D. Functionalized Polypropylenes: A Copolymerization and Postmodification Platform. *Macromolecules* **2024**, *57* (22), 10778-10791.

(5) Li, Y.; Yao, Z.; Chen, Z.-h.; Qiu, S.-l.; Zeng, C.; Cao, K. Rheological Evidence of Physical Cross-Links and Their Impact in Modified Polypropylene. *Ind. Eng. Chem. Res.* **2013**, *52* (23), 7758-7767.

(6) Cox, W. P.; Merz, E. H. Correlation of dynamic and steady flow viscosities. *J. Polym. Sci.* **1958**, *28* (118), 619-622.

(7) Gupta, S.; Yuan, X.; Mike Chung, T. C.; Cakmak, M.; Weiss, R. A. Influence of hydrogen bonding on the melt rheology of polypropylene. *Polymer* **2016**, *107*, 223-232.

(8) Stange, J.; Uhl, C.; Münstedt, H. Rheological behavior of blends from a linear and a long-chain branched polypropylene. *J. Rheol.* **2005**, *49* (5), 1059-1079.

(9) Fineman, M.; Ross, S. D. Linear method for determining monomer reactivity ratios in copolymerization. *J. Polym. Sci.* **1950**, *5* (2), 259-262.

(10) Kissin, Y. V.; Rishina, L. A.; Vizen, E. I. Hydrogen effects in propylene polymerization reactions with titanium-based Ziegler–Natta catalysts. II. Mechanism of the chain-transfer reaction. *J. Polym. Sci., Part A: Polym. Chem.* **2002**, *40* (11), 1899-1911.

(11) Tait, P. J. T.; Berry, I. G. 4 - Monoalkene Polymerization: Copolymerization. In *Comprehensive Polymer Science and Supplements*, Allen, G., Bevington, J. C. Eds.; Pergamon, 1989; pp 575-584.

(12) Uozumi, T.; Soga, K. Copolymerization of olefins with Kaminsky-Sinn-type catalysts. *Die Makromolekulare Chemie* **1992**, *193* (4), 823-831.
